# Simultaneous Presence of Mycotoxins in Feed Intended for Food-Producing Animals

**DOI:** 10.3390/foods14183176

**Published:** 2025-09-12

**Authors:** Loredana Annunziata, Guido Campana, Maria Rosaria De Massis, Giampiero Scortichini, Pierina Visciano

**Affiliations:** 1 Istituto Zooprofilattico Sperimentale dell’Abruzzo e del Molise “G. Caporale”, 64100 Teramo, Italy; 2 Department of Bioscience and Technology for Food, Agriculture and Environment, University of Teramo, 64100 Teramo, Italy

**Keywords:** aflatoxins, deoxynivalenol, ochratoxin, fumonisins, T-2 and HT-2 toxins, zearalenone, LC-MS/MS

## Abstract

This study aimed to verify the simultaneous presence of mycotoxins in feed intended for food-producing animals. A validated liquid chromatography–mass spectrometry analytical method was used for the determination and quantification of aflatoxins (AFB1, AFB2, AFG1, AFG2), deoxynivalenol, ochratoxin A, fumonisins, T-2 and HT-2 toxins, and zearalenone. The correlation coefficient indicated a good fit for all analytes, ranging from 0.991 to 0.999, while the mean recoveries were between 76 and 108%. The occurrence of one or more mycotoxins was detected in 42% of all feed samples investigated, at concentrations ranging between 0.0030 and 0.042 mg/kg for AFB1 and 0.16 and 0.95 and 0.016 and 1.5 mg/kg for deoxynivalenol and zearalenone, respectively. The sum of T-2 and HT-2 toxins ranged from 0.011 to 0.088 mg/kg, while the sum of fumonisins was between 0.010 and 14 mg/kg. Twenty-four positive samples (28%) showed the co-presence of ZEA and/or DON with FB1 and FB2, six of which were also contaminated with T-2 and HT-2 toxins. The need for continuous monitoring is particularly emphasized to ensure the health of both animals and humans.

## 1. Introduction

Feed can contain numerous undesirable substances that may pose a danger not only to animals but also to humans, due to their potential transfer into derived food products. Directive 2002/32/EC states that the use of feed intended for food-producing animals is prohibited when the presence of such compounds exceeds the maximum levels [1]. Among mycotoxins, the maximum content has been established only for aflatoxin B1 (AFB1) and corresponds to 0.02 mg/kg in feed materials and compound feed and to 0.01 mg/kg in complementary and complete feed. A lower limit of 0.005 mg/kg is mandatory for compound feed intended for dairy ruminants (cattle, sheep, and goats) [2] due to carry-over leading to the formation of aflatoxin M1 (AFM1) in milk collected from these animals fed with feedstuffs contaminated by AFB1 [3]. In contrast, for other mycotoxins, namely ochratoxin A (OTA), deoxynivalenol (DON), zearalenone (ZEA), fumonisins (FBs), and T-2 and HT-2, only guideline values are recommended because of their very limited transfer from feed to meat, milk, and eggs. Therefore, foods of animal origin contaminated with these mycotoxins contribute marginally to human exposure [4,5].

Mycotoxin-producing fungi can contaminate feed and food, with health implications for animals and humans, respectively. The toxicity of mycotoxins varies depending on their chemical structure [6]. Aflatoxins (AFs) are the most toxic compounds, showing hepatotoxic, immunotoxic, mutagenic, carcinogenic, and teratogenic effects in both animals and humans [7]. According to the International Agency for Research on Cancer (IARC), AFB1 and AFM1 are classified as carcinogenic to humans (Group 1) [8]. OTA exerts various adverse effects in animals, such as reduced body and/or organ weight, histopathological lesions, especially in the kidneys, immunotoxicity, neurotoxicity, and developmental damage [9]. Possible associations between OTA exposure and kidney disease as well as bladder and hepatocellular cancer have also been reported in humans [10]. It has been classified by IARC in Group 2B as a possible carcinogen [11]. DON can induce vomiting, anorexia, and other intestinal disorders in several animal species, as well as in humans [12]. It exhibits cellular toxicity effects such as mitochondrial dysfunction and oxidative stress, particularly on hepatocytes. Furthermore, it can directly suppress protein synthesis in humans [13]. ZEA has a similar structure to estrogens and thus competes for receptor binding, subsequently causing fertility and other reproductive disorders in livestock [14]. In humans, it is suspected to be the trigger for hyperestrogenism and early pubertal development in girls [15]. Among FBs, fumonisin B1 (FB1) is the most dangerous due to it causing hepatotoxicity, neurotoxicity, nephrotoxicity, immunotoxicity, developmental toxicity and cancer in both animals and humans [16]. FBs are also classified as 2B by IARC [8]. Further mycotoxins belonging to the trichothecene group for which maximum levels in food have recently been defined are T-2 and HT-2 toxins [17]. Hematotoxicity and immunotoxicity are the most critical effects of T-2, which is rapidly metabolized into HT-2, and therefore, its maximum content is referred to as their sum [18].

Since the co-occurrence of mycotoxins in crops and animal feed represents a huge challenge in terms of reduced animal performance and increased production costs [19], their simultaneous detection is particularly recommended [20]. Regulation (EU) 625/2017 requires each Member State to prepare a multiannual national control plan to ensure that official controls are planned in the areas of food and feed safety [21]. In Italy, the national official control plan on animal nutrition is published by the Ministry of Health and aims to ensure feed safety and quality. Particular attention should be paid in certain circumstances, such as when the administered feed is loaded with mold or the climatic conditions are optimal for fungal growth, but also when substantial changes in production performance and health status are observed in a large percentage of animals, which can be assumed as typical signs of mycotoxin effects [22]. The aim of this study was to monitor the simultaneous presence of several mycotoxins in animal feed using an in-house validated liquid chromatography–mass spectrometry (LC-MS/MS) method to assess compliance with regulatory maximum limits and/or guidance values established for feed.

## 2. Materials and Methods

### 2.1. Sampling Plan

Samples (*n* = 204) were collected from agricultural and livestock farms (*n* = 149) or feed manufacturing companies (*n* = 55) located in different regions of Central and Southern Italy throughout the years 2023–2024. The origin of the samples was linked to the territory under the jurisdiction of the competent authorities for official controls. They were divided into feed materials (*n* = 89) which are used directly as such or after processing and compound feed, i.e., mixtures of feed materials intended for oral feeding of animal as complete feed (*n* = 48) or complementary feed (*n* = 67). The difference between the latter two categories is related to their composition, since complete feed is sufficient for a daily ration, while complementary feed can only be used in combination with other feeds [1]. Sampling was carried out by the competent authorities according to the national plan of official controls on animal nutrition [22]. Specifically, it is based on the Italian database of livestock farms, their geographical distribution by region, and the results of monitoring plans from previous years. The criterion used is random sampling, aimed at assessing the epidemiological situation. All sampling data are reported as Supplementary Material (Table S1). The target analytes were AFB1, AFB2, AFG1, AFG2, DON, FB1, FB2, OTA, T-2 and HT-2 toxins, and ZEA.

### 2.2. Mycotoxin Analysis

#### 2.2.1. Reagents and Materials

All LC-MS/MS reagents, consisting of acetonitrile, acetic acid, formic acid, water, ammonium acetate, ammonium formate, and methanol, were ULC-MS-grade and purchased from Biosolve Chimie (Dieuze, France). Anhydrous magnesium sulfate (MgSO_4_) was obtained from Merck (Darmstadt, Germany). C_18_ silica sorbent (17% C loading, 40–63 µm, 60A) for dispersive solid-phase extraction was supplied by Silicycle (Quebec City, QC, Canada).

#### 2.2.2. Standards and Internal Standards

Individual standard solutions (AFB1, AFB2, AFG1, AFG2, DON, FB1, FB2, OTA, T-2 and HT-2 toxins, ZEA) and relative isotopically internal standards ([^13^C_17_]-AFB1, [^13^C_17_]-AFB2, [^13^C_17_]-AFG1, [^13^C_17_]-AFG2, [^13^C_15_]- DON, [^13^C_34_]-FB1, [^13^C_34_]-FB2, [^13^C_20_]-OTA, [^13^C_24_]-T-2 toxin, [^13^C_22_]-HT-2 toxin, [^13^C_15_]-ZEA) were purchased from RomerLabs (Getzersdorf, Austria).

#### 2.2.3. Preparation of Standard Solutions and Internal Standard Solutions

Working standard solutions were prepared at different concentrations: 100 ng/mL for AFB1, AFB2, AFG1, and AFG2, 500 ng/mL for T-2 and HT-2 toxins, 100 ng/mL for OTA, 5 µg/mL for ZEA, 10 µg/mL for DON, and 5 µg/mL for FB1 and FB2.

For isotopically internal standards, the following working solutions were prepared: 100 ng/mL for [^13^C_17_]-AFB1, [^13^C_17_]-AFB2, [^13^C_17_]-AFG1, [^13^C_17_]-AFG2, 500 ng/mL for [^13^C_24_]-T-2 toxin, [^13^C_22_]-HT-2 toxin, 100 ng/mL for [^13^C_20_]-OTA, 10 µg/mL for [^13^C_15_]-ZEA, [^13^C_15_]- DON and [^13^C_34_]-FB1, [^13^C_34_]-FB2.

#### 2.2.4. Sample Preparation

The sample was prepared according to Nualkaw et al. [23]. Briefly, extraction of 1 g of ground feed was performed using 10 mL of 1% formic acid aqueous solution and 10 mL of acetonitrile in a 50 mL polypropylene tube. After shaking, 1 g of NaCl and 4 g of MgSO_4_ were added to the extract. The tube was centrifuged for 5 min at 5000 rpm. After filtration through a PVDF filter, 2 mL of extract was placed in a 15 mL polypropylene tube and 0.1 g of C_18_ silica sorbent and 0.3 g of MgSO_4_ were added. After centrifugation for 5 min at 5000 rpm, the extract was dried under a nitrogen flow at 40 °C and resuspended with 1 mL of 20% methanol. After filtration through a PVDF filter, the sample was analyzed using LC-MS/MS.

#### 2.2.5. LC-MS/MS Analysis

Instrumental analysis was performed according to Nualkaw et al. [23] with some modifications. The same system and conditions were applied for chromatographic separation. For mass detection, a QTrap 6500^+^ system with a Turbo Spray IonDrive (Sciex, Framingham, MA, USA) was used in positive and negative (for DON and ZEA only) ionization modes operating in multiple reaction monitoring (MRM), selecting one precursor ion and two product ions for each analyte. The analytical system was managed by Analyst-SCIEX OS version 1.7.2 software.

The authors applied the instrumental conditions reported by Nualkaw et al. [23] for all mycotoxins, including DON and ZEA. The optimization of MS parameters was carried out by infusing individual solutions of analytes at a concentration of 1 mg/mL in 5 mM ammonium formate with 0.1% formic acid in water and methanol (50/50, *v*/*v*) for positive ionization mode. For negative ionization mode, individual solutions of analytes at a concentration of 1 mg/mL were infused in 5 mM ammonium acetate with 0.1% formic acid in water and methanol (50/50, *v*/*v*). The individual MRMs with their transition parameters in positive and negative ionization modes are shown in Table 1 and Table 2, respectively.

For positive ionization, the ion source temperature was set to 500 °C, the capillary voltage to 5100 V, the curtain gas to 35 °C, and the entrance potential to 10, while for negative ionization, the capillary voltage was set to −4300 V and the entrance potential to −10.

### 2.3. Method Validation

The method was validated according to the performance criteria established by the EUR-MP guidance document [24]. The parameters taken into account were instrumental linearity, limit of detection (LOD), limit of quantification (LOQ), precision, trueness, and uncertainty. Instrumental linearity was evaluated using solvent calibration curves at six different concentrations for each mycotoxin. Furthermore, 10 blank animal feeds for different animal species (chicken, cattle, pigs) were analyzed to evaluate specificity, LOD and LOQ. The estimation of LOD and LOQ was calculated according to Wenzl et al. [25]. The values obtained for LOD and LOQ were, respectively, 0.0007–0.0025 mg/mL for AFB1, AFB2, and AFG1; 0.0003–0.0010 mg/mL for AFG2; 0.046–0.16 mg/mL for DON; 0.004–0.015 mg/mL for FB1; 0.0015–0.0075 mg/mL for FB2; 0.0013–0.0050 mg/mL for OTA; 0.0016–0.0060 mg/mL for T-2; 0.0022–0.0080 mg/mL for HT-2; and 0.0040–0.015 mg/mL for ZEA. For repeatability and reproducibility, blank feed samples were spiked at four different concentration levels defined based on the established maximum content for AFB1 and the recommended guidance values for other mycotoxins. For FBs only, the spiked levels were equal to 1/10 of the guidance values. For each spiked level, three replicates were analyzed by two different operators, on two different occasions, for a total of 24 analyses. Trueness was expressed as recovery (percentage of the measured concentration compared to the fortified concentration) and precision as relative standard deviation (%RDS). According to the EURL-MP guidance, trueness can be assessed using certified reference materials or spiked samples. The method was validated with samples spiked at four different concentrations. Indeed, certified reference materials do not always contain all the analytes studied simultaneously and are not always commercially available. Accuracy and precision were assessed using matrix-matched standard calibration curves (Table 3) consisting of a blank sample subjected to the extraction procedure and fortified with mycotoxins and relative internal standards just prior to injection to obtain the spiked levels evaluated for the repeatability and reproducibility studies. From these two different validation days, the measurement uncertainty (MU) of the method was also determined according to the Nordtest Report [26] and expressed as expanded uncertainty. Statistical analysis was conducted using GraphPad InStat version 3.0.

## 3. Results and Discussion

### 3.1. Method Validation Results

Instrumental linearity was assessed for all mycotoxins at six calibration points (Table 4). Linearity was estimated using the least squares regression line equation. Solvent calibration curves were constructed using the ratio of analyte peak area to internal standard peak area versus analyte concentration. The correlation coefficient indicated a good fit for all mycotoxins with values in the range of 0.996–0.999. To establish method specificity, 10 blank feed samples were analyzed. All blank samples showed no interfering peaks at the retention time of interest for all analytes. Matrix-matched calibration curves were constructed ad hoc for each mycotoxin at six different concentration levels close to the expected LOD values (Table 5).

Because certified reference material was not available, trueness was assessed using recovery experiments. Repeatability and within-laboratory reproducibility were determined by analyzing blank samples spiked at four levels on two different days. To minimize matrix effects due to a straightforward sample preparation protocol, quantification was performed with matrix-matched standard calibration curves, plotting the analyte area divided by the area of internal standards versus the analyte concentration. The correlation coefficient indicated a good fit for all analytes, with values reported in the range of 0.991–0.999. Precision was expressed in terms of repeatability and within-laboratory reproducibility as RDS. The results of the precision study are summarized in Table 6.

Average recoveries for all investigated mycotoxins ranged between 76 and 108%. The maximum value of within-laboratory reproducibility was 12.6% at a spiking level of 0.010 mg/kg. Measurement uncertainty was estimated by considering the internal laboratory reproducibility. The value obtained according to the NT approach was multiplied by a coverage factor of 2 to obtain an expanded uncertainty calculated at a 95% confidence level (Table 6). The obtained uncertainty values ranged between 4 and 28%, 50% less than the value allowed by the EUL-MP guidance document [24].

### 3.2. Mycotoxin Occurrence in Feed Samples

The presence of one or more mycotoxins was detected in 85 (42%) samples, of which 35 (41%) were feed materials, 36 (42%) complementary feed, and 14 (17%) complete feed. The mean concentrations in the different categories of feed samples are reported in Table 7. All results are provided as Supplementary Material (Table S2). Twenty-four (28%) positive samples showed the co-occurrence of ZEA and/or DON with FB1 and FB2, six of which were also contaminated with T-2 and HT-2 toxins at concentrations ranging from 0.011 to 0.088 mg/kg. This latter group of mycotoxins was found alone in nine other samples and simultaneously with the sum of FB1 and FB2 in two samples. DON and ZEA concentrations ranged from 0.16 to 0.95 mg/kg and from 0.016 to 1.5 mg/kg, respectively. The sum of FB1 and FB2 ranged between 0.010 and 14 mg/kg. AFB1, AFG1 and AFG2 were detected in only 5.8, 2.4 and 7.1% of positive samples, respectively. Only one sample with AFB1 levels of 0.042 mg/kg did not comply with the regulatory limit of 0.020 mg/kg for feed materials. No sample exceeded the established guideline values for the other mycotoxins investigated. The presence of AFB1 is particularly concerning because it can be converted into AFM1 in the milk of dairy animals, resulting in human exposure to this mycotoxin. Additionally, low levels of mycotoxins in animal diets can lead to several disorders, such as slight decreases in feed intake and nutrient utilization, which can impact the production performance. Growth retardation, impaired immunity, and reduced disease resistance have also been reported [27].

Regarding the statistical analysis, the Kolmogorov–Smirnov test did not reveal any adherence to a normal distribution; then, a nonparametric Kruskal–Wallis test and Dunn’s multiple comparisons post-test were performed, comparing selected pairs of feed sample columns. A statistically significant difference was observed between feed materials and complementary feed for ZEA (*p* < 0.05), FB1, and the sum of FB1 and FB2 (*p* < 0.01). Sampling variability between different locations may have influenced these results, as mycotoxin prevalence is highly sensitive to localized climatic and agronomic conditions. In our study, particular attention was paid to the fact that raw materials and complementary feed undoubtedly constituted a more critical risk factor than complete feed. A positive correlation could be assumed between climatic conditions and the increase in contamination by molds and their metabolites. High temperatures and humidity are the main environmental factors contributing to the development of fungal invasions, and optimal conditions often occur in tropical and subtropical areas. However, the effects of climate change, such as prolonged droughts, extreme rainfall, increased temperatures and carbon dioxide levels, are also impacting continental climates in European countries, including Italy. Regarding the cultivation of raw materials, good agricultural practice and pest management could be effective strategies to preserve crop quality and safety.

Further studies conducted in Italian regions have reported the presence of mycotoxins in feed. Sdogati et al. [28] reported that feed had a higher incidence (26%) of contaminated samples compared to feed materials (6%) in Central Italy. AFB1 was detected in 9.3% of feed samples at maximum levels of 0.1045 mg/kg, while ZEA and OTA were found in 25 and 0.7% of samples at maximum concentrations of 6.420 and 0.085 mg/kg, respectively. Franchino et al. [29] found that the highest percentage of contaminated samples from Southern Italy (Puglia and Basilicata) was related to T-2 and HT-2 toxins (>70%), followed by ZEA, OTA and FBs (<42.5%), AFs (35%) and DON (<22%). Non-compliant samples were related to DON (0.9%), AF (0.6%) and ZEA (0.5%). Ferrari et al. [30] reported that 5.7% of the feed materials analyzed in an eight-year survey conducted in Northern Italy was found to be non-compliant for AFB1.

Regarding the incidence of mycotoxin contamination worldwide, the highest number of positive samples for AFs, OTA and ZEA was reported in Asia, followed by Europe, while the latter was the first-ranking continent with the highest number of observations of the presence of DON, T2 and HT-2 and FBs in animal feed. These data can be associated with mild temperatures, high humidity and heavy rainfall that can occur in many European countries, favoring fungal growth and mycotoxin production. The mycotoxin prevalence was reported in the following rank order: DON (74%) > ZEA (70%) > FBs (65%) > AFs (59%) > T-2 and HT-2 (45%) > OTA (31%) [31].

**Figure 1:**
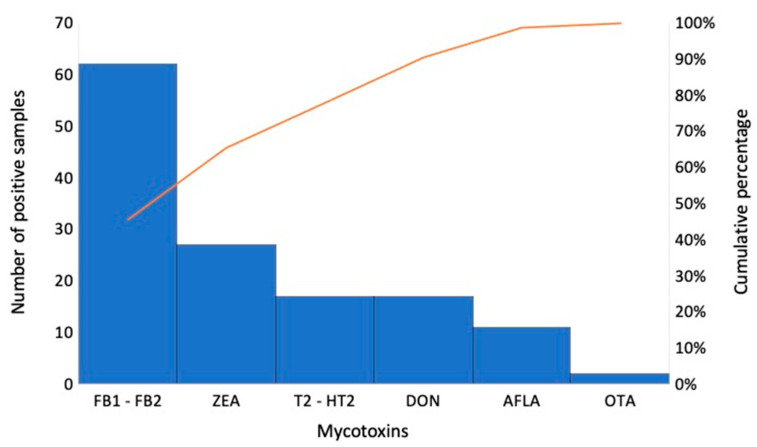
Pareto chart with a cumulative frequency line related to the investigated mycotoxins.

The incidence of each mycotoxin studied in all samples, regardless of feed category, is shown in Figure 1.

A similar trend in feed contamination by different groups of mycotoxins has been reported in the literature. Liu et al. [32] found incidence rates of 90.2, 77.4 and 33.9% for ZEA, DON and AFB1, respectively. The highest concentrations of ZEA and DON in maize samples were 1.6 and 1.3 mg/kg, while levels of 19 and 3.5 mg/kg were detected in wheat samples. The AFB1 maximum levels corresponded to 0.30 and 0.050 mg/kg, respectively. These high values were associated with the regions of origin of the samples, which experienced abundant rainfall and high temperatures during the sampling and analysis period. Kosicki et al. [33] reported that ZEA and DON were the most common occurring mycotoxins in 88 and 86% maize silage at maximum concentrations of 1.1 and 7.8 mg/kg, respectively. T-2 and HT-2 toxins were found in 47 and 73% of samples with maximum contents of 0.030 and 0.20 mg/kg, respectively. More than half of the samples (53%) were contaminated with FBs up to 0.10 mg/kg, while OTA was detected in 36% of the tested samples at a maximum value of 0.010 mg/kg. Nualkaw et al. [23] found that FBs, ZEA, DON and AFB1 were the most prevalent mycotoxins, contaminating feed samples up to concentrations of 26, 0.24, 1.4 and 0.32 mg/kg, respectively.

The presence of FBs (80.1 and 83.3%), DON (80.6 and 67.2%), ZEA (47.1 and 57.5%) and total AFs (9.6 and 5.8%) was observed in maize and finished feed from South Africa, respectively. The latter group exceeded the regulatory limit for AFB1 established in the European Union in 33.3% of maize samples and 54.4% of finished feed. OTA and T-2 toxin were detected at lower rates (7.4 and 3.1% and 0.7 and 1.3%, respectively). Maximum levels of FBs, DON and ZEA were found in maize and finished feed at concentrations of 16 and 7.5, 9.2 and 9.8, and 6.3 and 0.38 mg/kg, respectively. Total AFs and OTA showed maximum concentrations of 0.014 and 0.23 and 0.095 and 0.0060 mg/kg in maize and finished feed, respectively. The simultaneous presence of mycotoxins in the same sample was particularly related to FBs and DON (55.9%), FBs and ZEA (58.7%), and FBs and AFs (60.8%) [34].

**Figure 2:**
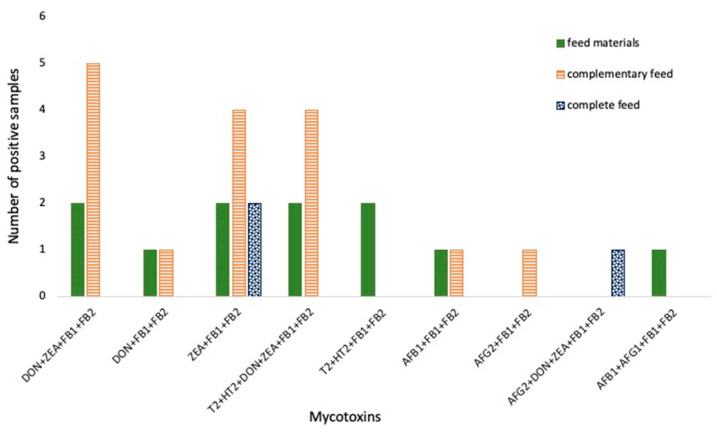
Co-occurrence of mycotoxins in feed samples.

In our study, 30 (35%) positive samples showed the co-occurrence of different groups of mycotoxins in the same feed sample (Figure 2).

The highest incidence was observed for DON + ZEA + FBs (23%), followed by the same mycotoxin groups with T-2 and HT-2, and ZEA + FBs (20%). The concomitant occurrence of DON and ZEA has been shown to be among the most common resulting from pre-harvest contamination by fungi of the *Fusarium* genus. They were simultaneously present in 93% of the forage maize samples examined by Birr et al. [35]. Abdallah et al. [36] reported that 61% of the feed samples were contaminated with DON, ZEA and FB1, and a further 21% showed the simultaneous presence of DON, ZEA, FB1, and T-2 toxin. Only 10% of the maize samples were found to be contaminated with both AFB1 and FB1. The maximum concentrations were 1.5, 2.4, 0.70, 0.030 and 0.010 mg/kg for DON, FB1, ZEA, T-2 toxin and AFB1, respectively.

Simultaneous exposure to multiple mycotoxins can cause more severe toxic effects in animals due to complex, often synergistic or additive interactions, which can exacerbate various disorders, such as immune suppression, impaired reproduction, oxidative stress and organ dysfunction, resulting in reduced growth performance, decreased milk and egg production and even increased mortality rates. For example, the concomitant presence of AFB1, OTA and ZEA can reduce milk yield in ruminants, while DON and ZEA together show synergistic action in pigs, causing reduced weight gain and feed intake. Immune and reproductive effects (i.e., immunosuppression and ovarian dysfunction) have been reported in pigs exposed to AFB1 and DON and ZEA and DON, respectively [37].

The prevalence of mycotoxin contamination in agricultural raw materials is associated with several factors, such as seasonality and climate conditions, agriculture management practices, and fungal ecology. Different fungi can produce mycotoxins both during harvest and post-harvest stages. Dimitrakopoulou et al. [38] demonstrated a greater seasonality impact on ZEA and DON than AF and OTA concentrations in animal feed. The authors also observed an inverse correlation between AFs and OTA levels, likely due to competitive interactions between fungal species. Conversely, the co-occurrence of ZEA and DON was likely associated with their production by the same *Fusarium* species that contaminated the feed, as also reported in further studies [39,40]. Regarding agronomic practices, nitrogen fertilization has been associated with high levels of ZEA and DON contamination in maize kernels, as well as an increased risk of other fungal metabolites produced by *Fusarium* species [41]. In contrast, crop rotation can interrupt the life cycle of mycotoxin-producing fungi, reducing the prevalence of mycotoxins such as DON and ZEA [42]. Balanced field irrigation is another agronomic measure reducing drought stress for plants that can consequently become more susceptible to infections, while excessive irrigation can lead to waterlogged conditions that favor the development of fungal species [43]. Correct planting density is also significant, as excessively dense crops can generate humid and poorly ventilated environments that enhance fungal growth [44]. Additionally, insect damage is a critical factor, as insects can create entry points for fungi and facilitate their spread in crops. Proper pest management and harvesting at the optimal time of crop maturity, minimizing mechanical damage, are essential to prevent the entry and growth of fungi. All these factors underline the importance of integrated agronomic practices to effectively control mycotoxin contamination [45]. For livestock and feed producers, key strategies may include feed analysis before feeding it to animals and proper drying of feed materials, combined with adequate storage conditions in well-ventilated facilities with controlled temperature and humidity [46].

Mycotoxin contamination in animal feed also represents a significant human health concern, as it can lead to the presence of mycotoxins in animal-derived products such as milk, posing a risk to consumers, particularly children and immunocompromised individuals. Carry-over of mycotoxins in milk depends on animal feed intake and milk production [47]. Some studies reported the presence of multiple mycotoxins in cow milk, such as AFs, FBs, ZEA, T-2 and HT-2 toxins, as well as emerging compounds, i.e., beauvericin and enniatin [48,49].

## 4. Conclusions

The present study revealed the co-presence of several mycotoxins in the same feed samples, although some groups were detected at low concentrations. No sample exceeded the guideline values for all mycotoxins, except one feed that was non-compliant for AFB1. These findings could pose a potential health risk to both animals and humans due to their possible synergistic and/or additive effects. Practical recommendations for farmers and feed producers could include pre- and post-harvest preventive measures, taking into account the variety of crop species with varying fungal resistance, and crop management (e.g., crop rotation, plowing, minimum-till or no-till technology, appropriate sowing date) in the former case, as well as cleaning and sorting practices and dehulling and thorough milling of grains after harvest. Thermal processes for reducing mycotoxins during feed production could be based on dry heating, superheated steam, extrusion cooking, and irradiation [50]. Since the current regulatory limits are related to individual mycotoxins and do not consider simultaneous exposure to multiple mycotoxins, a more holistic risk assessment approach that takes into account cumulative exposure and interaction effects should be applied [37].

Continuous monitoring of mycotoxins is strongly recommended to build a database of their presence in feed. The high-throughput LC-MS/MS analytical method used in this study for the determination and quantification of various mycotoxins in feed samples represents a valuable tool for achieving this important objective. Furthermore, risk assessment studies are needed for all groups of mycotoxins detected, even at low levels, to fully understand the potential adverse effects from human and animal exposure and to develop an effective risk mitigation plan. The mycotoxin concentrations detected in this study could be used to assess dietary exposure of animals based on consumption and species data.

## Figures and Tables

**Table 1:** LC-MS/MS parameters for detection in positive ionization mode.

Analyte	Rt	Precursor Ion (*m*/*z*)	Production (*m*/*z*)	DP (eV)	CE (eV)	CXP (eV)
AFB1	7.65	313.1	285.0	50	33	20
			241.0	50	51	20
IS-AFB1	7.65	330.1	255.1	70	50.3	13.5
AFB2	7.47	315.0	287.1	50	37	20
			259.0	50	41	24
IS-AFB2	7.47	332.1	303.2	70	36.8	25
AFG1	7.23	329.0	243.0	50	34	28
			200.0	50	51	22
IS-AFG1	7.23	346.2	212.1	70	54.2	18.1
AFG2	7.02	331.1	313.0	50	33	28
			285.2	50	38	27
IS-AFG2	7.03	348.1	259.2	70	43.6	16.1
FB1	8.55	722.0	352.0	50	49	26
			334.0	50	55	20
IS-FB1	8.54	756.5	356.4	83.7	55.9	17.5
FB2	9.25	706.0	336.0	50	53	16
			318.0	50	53	18
IS-FB2	9.25	740.6	358.4	87.3	50.7	23.2
T-2	8.61	484.0	215.0	69.9	25	14.8
			185.0	69.9	28.9	16.1
IS-T-2	8.61	508.3	229.2	77.6	26.7	16.8
HT-2	8.16	442.0	263.0	66.5	18.0	24
			215.0	66.5	18.6	24.5
IS-HT-2	8.16	464.3	229.1	70	18.1	17.5
OTA	8.99	404.0	239.0	80	30.9	21
			221.0	80	47.4	17
IS-OTA	8.99	424.2	250.1	85.1	32.3	21.4

Legend: Rt = indicative retention time; DP = declustering potential; CE = collision energy; CXP = collision cell exit potential.

**Table 2:** LC-MS/MS parameters for detection in negative ionization mode.

Analyte	Rt	Precursor ion (*m*/*z*)	Production (*m*/*z*)	DP (eV)	CE (eV)	CXP (eV)
DON	4.17	355.0	59.0	−58.7	−20.37	−17.09
			295.0	−58.7	−13.84	−8.34
IS-DON	4.17	370.4	310.1	−57.7	−14.37	−9.14
ZEA	9.07	317.0	175.0	−80	−32.59	−9.20
			131.0	−80	−38	−13
IS-ZEA	9.07	335.0	140.0	−80	−39.16	−7.28

Legend: Rt = indicative retention time; DP = declustering potential; CE = collision energy; CXP = collision cell exit potential.

**Table 3:** Matrix-matched standards calibration curves for precision study.

Analyte	Matrix-Matched Standard Calibration Curves (mg/kg)
AFB1	0.0025–0.0050–0.010–0.020
AFB2	0.0025–0.0050–0.010–0.020
AFG1	0.0025–0.0050–0.010–0.020
AFG2	0.0025–0.0050–0.010–0.020
DON	0.45–0.90–5.0–12
FB1	0.25–0.50–2.0–6.0
FB2	0.25–0.50–2.0–6.0
OTA	0.0050–0.010–0.10–0.25
T-2	0.0125–0.025–0.050–0.075
HT-2	0.0125–0.025–0.050–0.075
ZEA	0.050–0.20–0.50–3.0

**Table 4:** Solvent calibration curves.

Analyte	Calibration Curves in Solvent (ng/mL)
AFB1	0.125–0.25–0.50–1.0–2.0–4.0
AFB2	0.125–0.25–0.50–1.0–2.0–4.0
AFG1	0.125–0.25–0.50–1.0–2.0–4.0
AFG2	0.125–0.25–0.50–1.0–2.0–4.0
DON	22.5–45–200–500–800–1500
FB1	12.5–50–100–200–400–800
FB2	12.5–50–100–200–400–800
OTA	0.25–0.50–5–10–20–40
T-2	0.625–1.25–2.5–5.0–7.5–10
HT-2	0.625–1.25–2.5–5.0–7.5–10
ZEA	2.5–10–20–100–200–400

**Table 5:** Matrix-matched standards calibration curves for LOQ calculation.

Analyte	Matrix-Matched Standard Calibration Curves (mg/kg)
AFB1	0.00015–0.00030–0.00060–0.0012–0.0024
AFB2	0.00015–0.00030–0.00060–0.0012–0.0024
AFG1	0.00015–0.00030–0.00060–0.0012–0.0024
AFG2	00.00015–0.00030–0.00060–0.0012–0.0024
DON	0.0125–0.025–0.050–0.10–0.15
FB1	0.0075–0.015–0.030–0.045–0.060
FB2	0.0075–0.015–0.030–0.045–0.060
OTA	0.00030–0.00060–0.0012–0.0024–0.0048
T-2	0.0015–0.0030–0.0060–0.0090–0.012
HT-2	0.0015–0.0030–0.0060–0.0090–0.012
ZEA	0.0015–0.0030–0.0060–0.012–0.015

**Table 6:** Validation data.

Analyte	LOQ (mg/kg)	Spiking Levels (mg/kg)	Recovery (%) (Replicates *n* = 6)	Within-Reproducibility (Replicates *n* = 6)	MU (%)
AFB1	0.0025	0.0025	101	4.1	13
		0.0050	97	5.0	13
		0.010	85	6.1	18
		0.020	88	4.6	19
AFB2	0.0025	0.0025	99	9.9	21
		0.0050	96	12.1	21
		0.010	98	12.6	26
		0.020	98	7.3	26
AFG1	0.0025	0.0025	92	12.5	28
		0.0050	101	6.2	28
		0.010	95	7.7	17
		0.020	96	5.2	17
AFG2	0.0010	0.0025	101	5.0	11
		0.0050	105	5.9	11
		0.010	93	1.6	6
		0.020	100	2.9	6
DON	0.16	0.45	108	2.4	8
		0.90	106	2.8	7
		5.0	108	1.2	7
		10	105	1.0	7
FB1	0.015	0.25	99	6.0	13
		0.50	98	6.7	13
		2.0	93	7.7	16
		6.0	96	3.6	17
FB2	0.0075	0.25	102	5.8	12
		0.50	106	2.2	13
		2.0	102	3.6	8
		6.0	104	3.1	8
OTA	0.0050	0.0050	105	1.9	6
		0.010	104	1.1	6
		0.10	103	2.5	6
		0.20	99	2.8	6
T-2	0.0060	0.0125	106	2.9	8
		0.025	105	2.4	7
		0.050	107	1.3	6
		0.075	104	2.9	6
HT-2	0.0080	0.0125	99	9.1	19
		0.025	86	7.6	20
		0.050	94	12.0	25
		0.075	90	11.3	25
ZEA	0.015	0.050	102	1.3	13
		0.20	76	1.1	10
		0.50	102	1.0	4
		3.1	79	1.1	4

**Table 7:** Mycotoxin concentrations (mean ± S.D., mg/kg *) in feed sample categories.

Analyte	Feed Materials	Complementary Feed	Complete Feed
AFB1	0.018 ± 0.021	0.0031 **	0.0030 **
AFG1	0.0070 ± 0.0030	-	-
AFG2	0.020 ± 0.012	0.0021 **	0.0015 **
T-2	0.012 **	0.016 ± 0.0030	0.0083 **
HT-2	0.031 ± 0.036	0.028 ± 0.019	0.033 **
Sum T-2 and HT-2	0.034 ± 0.035	0.030 ± 0.025	0.041 **
OTA	-	0.038 ± 0.044	-
DON	0.57 ± 0.33	0.32 ± 0.21	0.30 ± 0.19
ZEA	0.25 ± 0.48 *^a^*	0.032 ± 0.019 *^b^*	0.035 ± 0.020
FB1	2.3 ± 3.3 *^A^*	0.19 ± 0.19 *^B^*	0.79 ± 1.3
FB2	0.58 ± 0.80	0.051 ± 0.051	0.45 ± 0.63
Sum FB1 and FB2	2.6 ± 3.9 *^A^*	0.23 ± 0.24 *^B^*	1.1 ± 1.9

Legend: S.D. = standard deviation; * concentrations calculated for a moisture content of 12%; ** single sample; *^a^*^, *b*^ (*p* < 0.05); *^A^*^, *B*^ (*p* < 0.01).

## Data Availability

The original contributions presented in this study are included in the article/Supplementary Materials. Further inquiries can be directed to the corresponding author.

## Supplementary Materials

The following supporting information can be downloaded at https://www.mdpi.com/article/10.3390/foods14183176/s1. Table S1: Sample categories, sampling site, province, and region of provenance. Table S2: Co-occurrence and concentrations (mg/kg ± MU) of mycotoxins in feed samples.
