# Supplementary material for: Simultaneous Presence of Mycotoxins in Feed Intended for Food-Producing Animals
Source: Foods. 2025 Sep 12;14(18):3176. doi: 10.3390/foods14183176 (PMC12469861; doi:10.3390/foods14183176)
Supplement: Supplementary file 1 [file foods-14-03176-s001.zip › foods-3855657-supplementary.pdf]

Table S1. Sample categories, sampling site, province, and region of provenance

Samples	Sampling site	Province	Region
Feed materials	Farm	L'Aquila	Abruzzo
Complete feed	Farm	Pescara	Abruzzo
Complementary feed	Farm	L'Aquila	Abruzzo
Complete feed	Farm	Chieti	Abruzzo
Complete feed	Farm	Chieti	Abruzzo
Complementary feed	Farm	L'Aquila	Abruzzo
Complementary feed	Farm	Pescara	Abruzzo
Complementary feed	Company	Pescara	Abruzzo
Complementary feed	Farm	Pescara	Abruzzo
Complementary feed	Company	Pescara	Abruzzo
Complementary feed	Company	Campobasso	Molise
Complementary feed	Company	Campobasso	Molise
Feed materials	Company	Bari	Puglia
Feed materials	Company	Matera	Basilicata
Feed materials	Farm	Potenza	Basilicata
Feed materials	Farm	Teramo	Abruzzo
Feed materials	Farm	Terni	Umbria
Feed materials	Farm	Ancona	Marche
Feed materials	Company	Bari	Puglia
Feed materials	Farm	Terni	Umbria
Feed materials	Company	Bari	Puglia

Feed materials	Farm	L’Aquila	Abruzzo
Feed materials	Farm	Perugia	Umbria
Feed materials	Company	Campobasso	Molise
Feed materials	Farm	Isernia	Molise
Feed materials	Farm	Isernia	Molise
Feed materials	Farm	Isernia	Molise
Feed materials	Farm	Foggia	Puglia
Feed materials	Farm	Campobasso	Molise
Feed materials	Company	Isernia	Molise
Feed materials	Farm	Isernia	Molise
Complete feed	Farm	Isernia	Molise
Complete feed	Company	Campobasso	Molise
Complete feed	Farm	Isernia	Molise
Complete feed	Company	Campobasso	Molise
Complete feed	Company	Matera	Basilicata
Complete feed	Farm	Teramo	Abruzzo
Complete feed	Company	Brindisi	Puglia
Complete feed	Company	Bari	Puglia
Complete feed	Farm	Isernia	Molise
Complete feed	Company	Campobasso	Molise
Complete feed	Company	Campobasso	Molise
Complete feed	Company	Campobasso	Molise
Complete feed	Company	Ancona	Marche

Complete feed	Company	Chieti	Abruzzo
Complete feed	Company	Teramo	Abruzzo
Complete feed	Farm	Teramo	Abruzzo
Complete feed	Company	Perugia	Umbria
Complete feed	Company	Perugia	Umbria
Complete feed	Company	Perugia	Umbria
Complete feed	Company	Campobasso	Molise
Complementary feed	Farm	Ascoli	Marche
Complementary feed	Farm	Ascoli	Marche
Complementary feed	Farm	Ascoli	Marche
Complementary feed	Farm	Potenza	Basilicata
Complementary feed	Company	Potenza	Basilicata
Complementary feed	Farm	Isernia	Molise
Complementary feed	Farm	Isernia	Molise
Complementary feed	Farm	Isernia	Molise
Complementary feed	Farm	Potenza	Basilicata
Complementary feed	Farm	Potenza	Basilicata
Complementary feed	Company	Pescara	Abruzzo
Complementary feed	Farm	Potenza	Basilicata
Complementary feed	Farm	Potenza	Basilicata
Complementary feed	Farm	Potenza	Basilicata
Complementary feed	Farm	Potenza	Basilicata
Complementary feed	Farm	Andria	Puglia

Complementary feed	Company	Campobasso	Molise
Complementary feed	Farm	Pescara	Abruzzo
Complementary feed	Farm	Pescara	Abruzzo
Complementary feed	Company	Pescara	Abruzzo
Complementary feed	Farm	Isernia	Molise
Complementary feed	Farm	Potenza	Basilicata
Complementary feed	Company	Campobasso	Molise
Complementary feed	Company	Potenza	Basilicata
Complementary feed	Company	Campobasso	Molise
Complementary feed	Farm	Matera	Basilicata
Complementary feed	Farm	Isernia	Molise
Complementary feed	Farm	L'Aquila	Abruzzo
Complementary feed	Company	Campobasso	Molise
Complementary feed	Farm	L'Aquila	Abruzzo
Complementary feed	Farm	Campobasso	Molise
Complementary feed	Farm	Isernia	Molise
Complementary feed	Farm	Perugia	Umbria
Complementary feed	Farm	L'Aquila	Abruzzo
Complementary feed	Farm	L'Aquila	Abruzzo
Complementary feed	Farm	L'Aquila	Abruzzo
Complementary feed	Farm	L'Aquila	Abruzzo
Complementary feed	Farm	L'Aquila	Abruzzo
Complementary feed	Company	Isernia	Molise

Complete feed	Farm	Isernia	Molise
Complete feed	Farm	L'Aquila	Abruzzo
Complete feed	Farm	L'Aquila	Abruzzo
Complete feed	Farm	Campobasso	Molise
Feed materials	Company	Bari	Puglia
Feed materials	Farm	Barletta	Puglia
Feed materials	Company	Pescara	Abruzzo
Feed materials	Company	Teramo	Abruzzo
Feed materials	Company	Teramo	Abruzzo
Feed materials	Company	Teramo	Abruzzo
Feed materials	Company	Pescara	Abruzzo
Feed materials	Company	Pesaro	Marche
Feed materials	Farm	Teramo	Abruzzo
Complete feed	Farm	L'Aquila	Abruzzo
Feed materials	Farm	L'Aquila	Abruzzo
Feed materials	Farm	L'Aquila	Abruzzo
Feed materials	Farm	L'Aquila	Abruzzo
Feed materials	Farm	L'Aquila	Abruzzo
Feed materials	Farm	L'Aquila	Abruzzo
Feed materials	Farm	L'Aquila	Abruzzo
Feed materials	Farm	L'Aquila	Abruzzo
Feed materials	Farm	L'Aquila	Abruzzo
Feed materials	Company	L'Aquila	Abruzzo

Feed materials	Farm	L’Aquila	Abruzzo
Feed materials	Farm	L’Aquila	Abruzzo
Feed materials	Farm	L’Aquila	Abruzzo
Feed materials	Farm	L’Aquila	Abruzzo
Feed materials	Farm	L’Aquila	Abruzzo
Feed materials	Farm	L’Aquila	Abruzzo
Feed materials	Farm	L’Aquila	Abruzzo
Feed materials	Farm	L’Aquila	Abruzzo
Feed materials	Farm	Teramo	Abruzzo
Feed materials	Farm	L’Aquila	Abruzzo
Feed materials	Farm	L’Aquila	Abruzzo
Feed materials	Farm	L’Aquila	Abruzzo
Feed materials	Farm	L’Aquila	Abruzzo
Feed materials	Farm	L’Aquila	Abruzzo
Feed materials	Farm	L’Aquila	Abruzzo
Feed materials	Farm	L’Aquila	Abruzzo
Feed materials	Farm	L’Aquila	Abruzzo
Feed materials	Farm	L’Aquila	Abruzzo
Feed materials	Farm	L’Aquila	Abruzzo
Feed materials	Farm	L’Aquila	Abruzzo
Feed materials	Farm	L’Aquila	Abruzzo
Feed materials	Farm	L’Aquila	Abruzzo
Feed materials	Farm	L’Aquila	Abruzzo

Feed materials	Farm	L’Aquila	Abruzzo
Feed materials	Farm	L’Aquila	Abruzzo
Feed materials	Farm	L’Aquila	Abruzzo
Feed materials	Farm	L’Aquila	Abruzzo
Feed materials	Farm	L’Aquila	Abruzzo
Feed materials	Farm	L’Aquila	Abruzzo
Feed materials	Farm	L’Aquila	Abruzzo
Feed materials	Farm	L’Aquila	Abruzzo
Feed materials	Farm	L’Aquila	Abruzzo
Feed materials	Company	Perugia	Umbria
Feed materials	Company	Pescara	Abruzzo
Complete feed	Company	Teramo	Abruzzo
Complete feed	Company	L’Aquila	Abruzzo
Complete feed	Farm	Isernia	Molise
Complete feed	Company	Teramo	Abruzzo
Complete feed	Farm	Teramo	Abruzzo
Complete feed	Farm	Chieti	Abruzzo
Complete feed	Farm	Chieti	Abruzzo
Complementary feed	Farm	Teramo	Abruzzo
Complementary feed	Company	Perugia	Umbria
Complementary feed	Farm	Pescara	Abruzzo
Complementary feed	Farm	Teramo	Abruzzo
Complementary feed	Farm	Perugia	Umbria

Complementary feed	Farm	Teramo	Abruzzo
Complementary feed	Farm	Pescara	Abruzzo
Complementary feed	Farm	Teramo	Abruzzo
Complementary feed	Farm	Chieti	Abruzzo
Complementary feed	Farm	Teramo	Abruzzo
Complementary feed	Farm	Chieti	Abruzzo
Complementary feed	Farm	Chieti	Abruzzo
Complementary feed	Farm	Chieti	Abruzzo
Complementary feed	Company	Chieti	Abruzzo
Complementary feed	Farm	Chieti	Abruzzo
Complementary feed	Farm	Chieti	Abruzzo
Complete feed	Company	Campobasso	Molise
Complete feed	Farm	L’Aquila	Abruzzo
Feed materials	Farm	Teramo	Abruzzo
Feed materials	Company	Taranto	Puglia
Feed materials	Farm	Pescara	Abruzzo
Feed materials	Farm	Ascoli	Molise
Complete feed	Farm	Teramo	Abruzzo
Feed materials	Farm	L’Aquila	Abruzzo
Feed materials	Farm	L’Aquila	Abruzzo
Feed materials	Farm	L’Aquila	Abruzzo
Feed materials	Farm	Teramo	Abruzzo
Feed materials	Company	Teramo	Abruzzo

Feed materials	Farm	L’Aquila	Abruzzo
Feed materials	Farm	L’Aquila	Abruzzo
Feed materials	Farm	Fermo	Marche
Feed materials	Company	Bari	Puglia
Feed materials	Farm	Pescara	Abruzzo
Feed materials	Farm	Pescara	Abruzzo
Feed materials	Farm	Pescara	Abruzzo
Feed materials	Farm	Pescara	Abruzzo
Complete feed	Farm	L’Aquila	Abruzzo
Complete feed	Farm	L’Aquila	Abruzzo
Complete feed	Farm	L’Aquila	Abruzzo
Complete feed	Company	Teramo	Abruzzo
Complete feed	Farm	L’Aquila	Abruzzo
Complete feed	Farm	L’Aquila	Abruzzo
Complete feed	Company	Chieti	Abruzzo
Complete feed	Farm	Macerata	Marche
Complete feed	Farm	Macerata	Marche
Complementary feed	Farm	Chieti	Abruzzo
Complementary feed	Farm	Chieti	Abruzzo
Complementary feed	Farm	Chieti	Abruzzo
Complementary feed	Farm	Chieti	Abruzzo
Complete feed	Farm	Pescara	Abruzzo

Table S2. Co-occurrence and concentrations (mg/kg* ± MU**) of mycotoxins in feed samples

Samples	AFB1	AFG1	AFG2	T-2	HT-2	Sum T-2/HT-2	OTA	DON	ZEA	FB1	FB2	Sum FB1 FB2
Complementary feed				0.016	0.039	0.055						
Complete feed				0.0083	0.033	0.041						
Complementary feed				0.014	0.052	0.066						
Complementary feed				0.020	0.068	0.088						
Complementary feed					0.023	0.023						
Complementary feed					0.013	0.013						
Complementary feed					0.012	0.012						
Complementary feed				0.013	0.042	0.055						
Complementary feed					0.029	0.029						
Feed materials								0.65	0.16	0.076	0.021	0.097
Feed materials								0.95	0.22	0.077	0.039	0.12
Feed materials										0.43	0.20	0.63
Feed materials										1.30	0.35	1.7
Feed materials									0.024			
Feed materials										0.22	0.044	0.26
Complete feed									0.021	0.027		0.027
Complete feed			0.0015					0.43	0.067	1.20	0.34	1.5
Complete feed										0.066	0.085	0.15
Complete feed									0.038			
Complete feed										4.1	1.8	5.9
Complete feed	0.0030											

Complete feed						0.034			
Complementary feed								0.037	0.037
Complementary feed					0.20	0.034	0.039		0.039
Complementary feed					0.26	0.021	0.076		0.076
Complementary feed			0.015	0.015	0.36	0.024	0.11	0.027	0.14
Complementary feed			0.013	0.013	0.17	0.019	0.40	0.093	0.49
Complementary feed			0.014	0.014		0.016	0.17	0.027	0.20
Complementary feed			0.012	0.012	0.16	0.027	0.57	0.13	0.70
Complementary feed		0.0021					0.016	0.0086	0.025
Complementary feed					0.87	0.022	0.021		0.021
Complementary feed						0.028	0.020	0.013	0.033
Complementary feed							0.21	0.061	0.27
Complementary feed							0.031	0.017	0.048
Complementary feed						0.025	0.16	0.021	0.18
Complementary feed							0.10	0.040	0.14
Complete feed						0.016	0.086	0.013	0.099
Feed materials						0.034	0.024	0.0079	0.032
Feed materials							2.7	1.0	3.7
Feed materials			0.011	0.011	0.32	0.091	3.7	1.3	5.0
Feed materials							0.19	0.023	0.21
Feed materials	0.0048	0.038							
Feed materials		0.011							
Feed materials		0.017							
Feed materials		0.015							
Feed materials						1.5 ± 0.10			
Feed materials							2.6	0.70	3.3
Complete feed							0.026		0.026

Complete feed										0.40	0.27	0.67
Complete feed										0.48	0.37	0.85
Complementary feed								0.31	0.033	0.092	0.024	0.12
Complementary feed										0.11	0.024	0.13
Complementary feed									0.042	0.16	0.057	0.21
Complementary feed										0.38	0.12	0.50
Complementary feed										0.020	0.0093	0.029
Complementary feed								0.20	0.030	0.41	0.086	0.50
Complementary feed										0.062	0.014	0.077
Complementary feed								0.27		0.64	0.085	0.72
Complementary feed										0.070	0.013	0.083
Complementary feed	0.0031									0.60	0.21	0.81
Complementary feed							0.0066					
Complete feed								0.16				
Feed materials										0.53	0.22	0.75
Feed materials									0.024	0.25	0.054	0.30
Feed materials										0.14	0.036	0.17
Feed materials											0.020	0.020
Feed materials											0.014	0.014
Feed materials					0.012	0.012				0.018	0.011	0.029
Feed materials				0.012	0.015	0.027					0.010	0.010
Feed materials									0.045			
Feed materials										9.4	2.6	12
Feed materials	0.0050									4.9	1.3	6.2
Feed materials	0.0079											
Feed materials	0.042 ± 0.019	0.0089								6.6	2.0	8.6
Feed materials								0.16		0.79	0.32	1.1

Feed materials	0.085	0.085		0.77	0.13	12	1.8	14
Feed materials						0.82	0.43	1.2
Feed materials						0.85	0.71	1.6
Feed materials						6.7	2.4	9.1
Feed materials						0.046	0.021	0.067
Feed materials						0.036	0.017	0.053
Complementary feed			0.069					
Complementary feed				0.37	0.091	0.070	0.016	0.086
Complementary feed						0.27		0.27
Complete feed						0.78	0.21	0.99

Legend: *concentrations calculated for a moisture content of 12%; **measurement uncertainty
